# Chronic coronary syndrome with suspected Takayasu’s arteritis

**DOI:** 10.1093/ehjimp/qyaf028

**Published:** 2025-03-13

**Authors:** Adriana Złahoda-Huzior, Maria Kundzierewicz, Katarzyna Kołodziej, Robert Banys, Jacek Godlewski, Dariusz Dudek, Małgorzata Urbańczyk-Zawadzka

**Affiliations:** Department of Measurement and Electronics, AGH University of Science and Technology, Al. Mickiewicza 30, 30-059 Krakow, Poland; SimHub, Virmed, Al. Plk. Wl. Beliny-Prazmowskiego 62/2, 31-514 Krakow, Poland; SimHub, Virmed, Al. Plk. Wl. Beliny-Prazmowskiego 62/2, 31-514 Krakow, Poland; SimHub, Virmed, Al. Plk. Wl. Beliny-Prazmowskiego 62/2, 31-514 Krakow, Poland; Clinical Research Center, Intercard, Krakow, Poland; Clinical Research Center, Intercard, Krakow, Poland; Center of Digital Medicine and Robotics, Jagiellonian University Medical College, Krakow, Poland; Maria Cecilia Hospital, GVM Care & Research, Cotignola, Italy; Clinical Research Center, Intercard, Krakow, Poland

**Keywords:** Takayasu's arteritis, computed tomography angiography, multi-modality imaging, percutaneous coronary intervention, cardiac imaging

A 54-year-old woman with chronic coronary artery syndrome and hypertension was referred to further hospital diagnosis from the outpatient clinic due to exertional angina. Patient had normal results of routine blood tests.

Invasive coronary angiography (ICA) revealed ostial stenoses of coronary arteries narrowing to 95% with no significant stenoses in remaining part of the vessels (*Figure 1A*). ICA raised the suspicion of Takayasu's arteritis—a systemic inflammation of the large vessels involving the aorta and its main branches. As its clinical presentation is often related to musculoskeletal symptoms and ischaemic manifestations, the patient was referred for further detail imaging diagnosis with computed tomography angiography (CTA).

**Figure 1:**
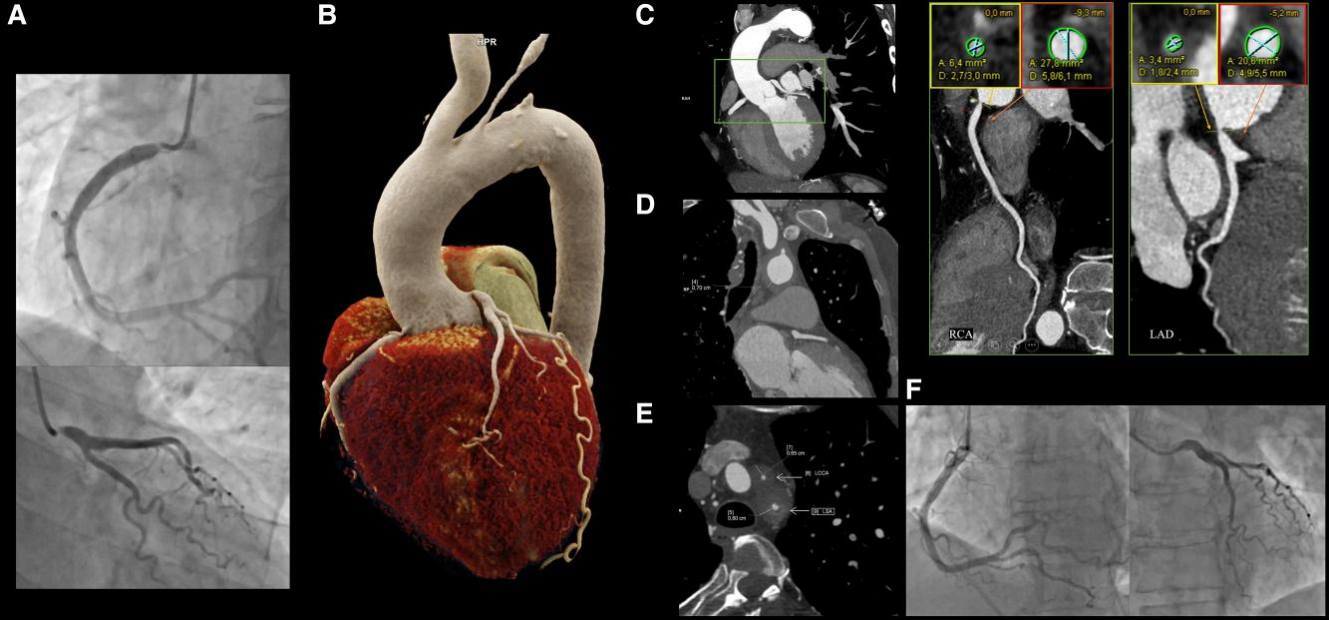
Imaging examinations. (*A*) ICA of right and left coronary arteries presenting ostial lesions. (*B*) Spatial volume rendering of CTA. Ostial lesions of coronary arteries are presented with total occlusion ofLSA and significant narrowing of LCCA. (*C*) Ostial lesions of coronary arteries presented in multiplanar reconstruction of CTA, including diameter measurements of stenosis and distal references for both coronaries. (*D*) Thickening of aortic wall up to 7 mm in CTA. (*E*) Narrowing of LSA and LCCA, with wall thickness 8 and 6 mm, respectively. (*F*) Final result of coronary angiography post-PCI.

CTA confirmed ICA findings (*Figure 1B*, see Supplementary data online, *Video S1*), presenting ostial stenosis for both coronary arteries with preserved vessel patency (*Figure 1C*). Aortic arch was covered with single calcified atherosclerotic plaques, with peripheral thickening of the wall around 4 mm—above the brachiocephalic trunk, up to 7–8 mm—at the level of the left common carotid artery (LCCA) (*Figure 1D*). Proximal part of LCCA wall was thickened up to 6 mm. The left subclavian artery lumen was visible up to 6 mm above the arch with wall thickness of 7–8 mm, gradually narrowing to total occlusion including left internal mammary artery (*Figure 1E*).

The patient underwent percutaneous coronary intervention (PCI) of the right coronary artery with implantation of a drug-eluting stent (DES) 4.5 × 12 mm. Intravascular ultrasound assessment confirmed proper stent apposition. Two months later, PCI of the left main coronary artery was performed, involving the implantation of a DES 5.0 × 12 mm. The procedure resulted in optimal angiographic outcomes and no complications (*Figure 1F*).

The patient in good condition was discharged home with recommendations for diet, medication, and ongoing cardiovascular follow-up.

## Supplementary Material

qyaf028_Supplementary_Data

## Notes

**Consent:** Patient consent was obtained prior publication.

**Funding:** None declared.

**Data availability:** No new data were generated or analysed in support of this research.

## Lead author biography

Adriana Zlahoda-Huzior is a biomedical engineer at AGH University of Science and Technology (AGH UST) in Krakow, Poland. She specializes in novel medical technologies and medical imaging, with a particular focus on cardiology.Her research interests lie at the intersection of advanced imaging techniques and their applications in cardiovascular health. Adriana is dedicated to exploring innovative imaging techniques and visualizations to enhance diagnostic accuracy and improve patient outcomes in cardiology, such as: Extended Realities, 3D modelling, 3D printing, and 3D data reporting.

Currently, she is engaged in several significant research projects aimed at developing and optimizing imaging modalities for better visualization of cardiac structures and functions prior to coronary and structural heart interventions. Through collaboration with interdisciplinary teams, she strives to contribute to the advancement of medical technologies.

Adriana has authored and co-authored numerous publications in reputable journals, which can be found on her Google Scholar profile. Her work reflects her commitment to advancing knowledge in the field of biomedical engineering and medical imaging.

In addition to her research, she is actively involved in professional affiliations and contributes to the academic community through presentations and workshops during international congresses such as *EuroPCR, TCT,* and *PCR London Valves.*

Looking ahead, Adriana aims to expand her research into emerging imaging technologies and their integration into clinical practice, ultimately enhancing the effectiveness of cardiological diagnostics and therapies.

